# Bioactivity of Grape Pomace Extract and Sodium Selenite, Key Components of the OenoGrape Advanced Complex, on Target Human Cells: Intracellular ROS Scavenging and Nrf2/ARE Induction Following In Vitro Intestinal Absorption

**DOI:** 10.3390/antiox13111392

**Published:** 2024-11-14

**Authors:** Cécile Dufour, Camille Gironde, Mylène Rigal, Christophe Furger, Erwan Le Roux

**Affiliations:** 1 Anti Oxidant Power, 78 Allées Jean Jaurès, 31000 Toulouse, France; cdufour@antioxidant-power.com (C.D.); cgironde@laas.fr (C.G.); mrigal@laas.fr (M.R.); 2 Cooper Consumer Health, Place Lucien Auvert, 77000 Melun, France

**Keywords:** cellular antioxidant assays, AOP1 assay, Nrf2/ARE pathway, efficacy, food supplement, cytoprotection, bioavailability, bioactivity, grape, selenium, lycopene

## Abstract

Oenobiol Sun Expert, a food formulation designed to enhance skin health prior to sun exposure, has been optimized by incorporating the OenoGrape Advanced Complex, which includes grape pomace extract, increased selenium content and 10% lycopene-rich tomato extract, with these constituents exhibiting high antioxidant potential. To evaluate the effects of these individual ingredients and the overall formulation at the cellular level, the AOP1 cell antioxidant efficacy assay was employed to measure the intracellular free radical scavenging activity, while the Cell Antioxidant Assay (CAA or DCFH-DA) assay was used to assess peroxidation scavenging at the plasma membrane level. The indirect antioxidant activity was examined using stably transfected cell lines containing a luciferase reporter gene controlled by the Antioxidant Response Element (ARE), which activates the endogenous antioxidant system via the Nrf2/Keap1-ARE pathway. Our results indicate that among the individual components, grape pomace extract and sodium selenite possess high and complementary antioxidant properties. Grape pomace extract was particularly effective in inhibiting free radicals (AOP1 EC_50_ = 6.80 μg/mL) and activating the ARE pathway (ARE EC_50_ = 231.1 μg/mL), whereas sodium selenite exerted its effects through potent ARE activation at sub-microgram levels (EC_50_ = 0.367 μg/mL). In contrast, the lycopene-rich tomato extract did not show a notable contribution to the antioxidant effects. The antiradical activity of the OenoGrape Advanced Complex, comprising these three ingredients, was very efficient and consistent with the results obtained for the individual components (AOP1 EC_50_ = 15.78 µg/mL and ARE EC_50_ of 707.7 μg/mL). Similarly, the free radical scavenging activity still persisted in the Oenobiol Sun Expert formulation (AOP1 EC_50_ = 36.63 µg/mL). Next, in vitro intestinal transepithelial transfer experiments were performed. The basolateral compartments of cells exposed to the ingredients were collected and assessed using the same antioxidant cell assays. The direct and indirect antioxidant activities were measured on both hepatocytes and keratinocytes, demonstrating the bioavailability and bioactivity of grape pomace extract and sodium selenite. These finding suggest that the ingredients of this food supplement contribute to enhanced cytoprotection following ingestion.

## 1. Introduction

Reactive Oxygen Species (ROS), including various radicals and non-radicals, along with other reactive species, such as Reactive Nitrogen Species (RNS), are generated in the human body from both intrinsic factors (cellular metabolism, time/aging and genetics) and extrinsic factors (visible/UV radiation, pollution, nutrition and lifestyle). At low levels, ROS play a role in cell signaling and survival. However, at elevated levels, these highly reactive species contribute to the accumulation of macromolecular damage—impacting lipids, proteins and DNA—and are associated with an increased risk of developing chronic diseases [1,2,3]. Human cells possess an antioxidant system capable of neutralizing oxidative species, thereby maintaining cellular homeostasis, which is essential for physiological functions [4]. Consequently, balancing oxidants and antioxidants within a constantly changing environment is critical for health. Both endogenous and exogenous antioxidants are important for the defense against excessive ROS production. Vitamins, carotenoids and polyphenolic compounds, which humans cannot synthesize, represent potent natural antioxidants. In certain circumstances, such as inadequate dietary intake, a demanding lifestyle or compromised defense mechanisms (during exercise, sun exposure, chronic diseases or aging), supplementation with the micronutrients or essential cofactors that are involved in redox reactions (vitamins, metal ions and trace elements) may be necessary.

Antioxidant molecules, also referred as reductants, function by donating electrons to oxidant compounds (electron acceptors) in redox reactions. However, the definition of “antioxidant” is broad. In chemistry, antioxidants are radical scavengers that interrupt radical chain reactions involving oxygen and other substrates, while in cell biology and medicine, antioxidants refer to enzymes or organic substances that are capable of counteracting the harmful effects of ROS and RNS on physiological processes. Dietary antioxidants can neutralize ROS/RNS and prevent the formation of reactive species or act as metal chelators, oxidative enzyme inhibitors and cofactors for antioxidant enzymes. In response to oxidative stress, living cells have evolved, and indirect antioxidant mechanisms, such as the Nrf2-Keap1-ARE pathway, can enhance the cell’s antioxidant capacity through the transcriptional induction of genes encoding antioxidant and cytoprotective enzymes [5,6,7]. Therefore, antioxidant compounds engage in multiple biochemical reactions to maintain cell redox homeostasis. However, these effects are not measurable using traditional antioxidant assays (e.g., ORAC and DPPH) conducted in cell-free systems, as these tests target only two main categories of chemical reactions: hydrogen atom transfer (HAT) and electron transfer (ET) [8,9]. Despite the extensive literature supporting these assays, their limitations have undermined the credibility of antioxidants, as their biological activities could not be accurately determined in test tubes. In 2012, the USDA removed the ORAC database from its website, citing “mountain evidence that the values indicating antioxidant capacity have no relevance to the effects of specific bioactive compounds, including polyphenols, on human health” (as cited in [10]).

It is now essential to integrate standardized in vitro cell-based assays to measure the antioxidant activity and elucidate the mechanisms of action at the cellular level, in a complex environment, to clarify the effects of antioxidants and characterize the bioactivity of specific supplement components. Natural botanical extracts and nutraceuticals typically contain multiple bioactive compounds. In many cases, data on individual bioactives are extrapolated to combined components of marketed nutraceuticals without testing the final formulation. Synergic effects from interacting bioactive molecules can be demonstrated using in vitro cell-based assays [11]. It is also important to note that excessive antioxidant supplementation can disturb redox signaling, inducing reductive stress or leading to ROS generation with pro-oxidant and cytotoxic effects. Thus, in vitro cell-based assays enable precise dose-dependent evaluations of antioxidant activity and efficacy concentrations, providing a starting point for bioactivity assessments. These preclinical tests may inform in vitro–in vivo correlation studies for robust clinical trials, increasing the probability of detecting physiological effects.

Oenobiol Sun Expert is a newly formulated version of Oenobiol Solaire Intensif, designed to enhance skin defenses against oxidative stress from sun exposure. The formulation comprises the OenoGrape Advanced Complex, which consists of grape pomace extract, an increased selenium content (30% higher) and 10% lycopene-rich tomato extract, due to their high antioxidant potentials. This study aimed to (1) quantify the antioxidant properties using a set of in vitro cell-based assays and (2) investigate the bioavailability and bioactivity. In vitro intestinal transepithelial transfer experiments were performed, basolateral compartments were collected, and the antioxidant activities of the transported compounds and/or metabolites were assessed on target cells.

## 2. Materials and Methods

### 2.1. Chemicals and Cell Lines

Sulforaphane (SFN), thiazole orange (TO), 2,2-azobis(2-methylpropionamidine) dihydrochloride (AAPH) and 2′,7′-dichlorofluorescin diacetate (DCFH-DA) were obtained from Sigma-Aldrich (Saint-Quentin Fallavier, France). Gibco DMEM (high glucose, GlutaMAX supplement and pyruvate), fetal bovine serum (FBS) (HyClone), pen-strep solution (100X) (Gibco), 0.05% Trypsin-EDTA (HyClone), Gibco Selective Antibiotic Geneticin (G418) (50 mg/mL) and Gibco DPBS without Calcium and Magnesium (1X) were procured from Thermo Fisher Scientific (Illkirch-Graffenstaden, France). Additionally, 12 mm Transwell permeable supports with 3 μm pore polycarbonate membrane inserts (3402, Corning) were purchased from Thermo Fisher Scientific (Illkirch-Graffenstaden, France). The HepG2 cell line (catalogue number HB8065) was obtained from the American Type Cell Collection (ATCC) (LGC Standards, Molsheim, France), while the Caco2 and HaCaT cell lines were kindly provided by Led Engineering Development (LED, Montauban, France). The ARE Reporter–HepG2 cell line (catalogue number 60513) was acquired from BPS Bioscience (San Diego, CA, USA), and the ARE Reporter–HaCaT cell line was a generous gift from A. Natsch.

### 2.2. OenoGrape Advanced Complex, Oenobiol Sun Expert and Oenobiol Solaire Intensif Formulations

The ingredients for the formulations were sourced from Cooper Consumer Health (Melun, France). The composition per capsule of the Oenobiol Solaire Intensif formulation is as follows: 115 mg of tomato extract (equivalent to 8 mg of lycopene), 0.050 mg of selenium, 10 mg of vitamin E, 5.25 mg of lutein and 0.95 mg of vitamin B2. The total capsule weight was 451 mg. The composition per capsule of the Oenobiol Sun Expert formulation is as follows: 100 mg of tomato extract (equivalent to 10 mg of lycopene), 60 mg of grape pomace extract, 0.065 mg of selenium, 10 mg of vitamin E (from a natural extract), 5.25 mg of lutein, 0.15 mg of cooper and 0.7 mg of vitamin B2. The total capsule weight was 450 mg. The OenoGrape Advanced Complex was composed of the following: 100 mg of tomato extract (equivalent to 10 mg of lycopene), 60 mg of grape pomace extract and 0.065 mg of selenium (% (m/m)), corresponding to 62.44% of tomato extract, 37.47% of grape pomace extract and 0.041% of anhydrous sodium selenite.

### 2.3. Preparation of Ingredients and Formulations for Antioxidant Cell-Based Assays

For the preparation of grape pomace extract and sodium selenite, DMEM without serum was added to the weighed powders to achieve final concentrations of 50 mg/mL. The preparations were vortexed thoroughly and centrifuged at 8700 rpm for 10 min. The supernatants were aliquoted and stored at −20 °C for the subsequent cell-based assays. For the preparation of the 10% lycopene-rich tomato extract, the sample was mixed with DMEM without serum and sonicated. The sample concentrations were determined based on the dry matter weight. The OenoGrape Advanced Complex was prepared according to the proportions described in the Oenobiol Sun Expert formulation, with DMEM without serum added to reach a final concentration of 50 mg/mL. The mixture was vortexed and centrifuged, and the supernatant was aliquoted and stored at −20 °C for use in cell-based assays. For the preparation of the Oenobiol Sun Expert and Solaire Intensif formulations, ethanol was used as solvent to solubilize both formulations in DMEM without serum, also aiming for a final concentration of 50 mg/mL. The solutions were vortexed and centrifuged, and the resulting supernatants were stored in aliquots at −20 °C. For the dose–response studies, a series of decreasing concentration ranges (C1max to C9) were prepared from the stored aliquots using serial dilutions, allowing for a 3-log range evaluation. All concentrations and the use of organic solvents, if applicable, are indicated in the corresponding figures and legends.

### 2.4. Cell Culture Conditions

Human hepatocytes from the HepG2 cell line (passages 15 to 35) and human keratinocytes from the HaCaT cell line (passages 10 to 40) were cultured at 37 °C with 5% CO_2_ in GlutaMAX-supplemented DMEM complemented with 10% FBS and 1% penicillin–streptomycin (1X) solution. ARE Reporter–HepG2 cells (passages 3 to 16) and ARE Reporter–HaCaT cells (passages 10 to 21) were cultured under the same conditions with the addition of 0.6 mg/mL of Geneticin to the medium. Human enterocyte-like cells from the Caco2 cell line (passages 20 to 40) were cultured at 37 °C with 5% CO_2_ in GlutaMAX DMEM complemented with 20% FBS and 1% pen-strep (1X). Once the cells reached 70–80% confluence, they were transferred into clear-bottom 96-well microplates for 24 h: HepG2 cells were plated at 75,000 cells/well, and HaCaT cells were plated at 40,000 cells/well (75 μL/well). The Caco2 cells were transferred into 12-well microplates with Transwell inserts at 760,000 cells/Transwell (500 µL/Transwell).

### 2.5. Intracellular ROS Scavenging Bioassay with AOP1 Assay

The AOP1 bioassay (patented technology) measures the ability of compounds to scavenge intracellularly generated reactive species using a photosensitive biosensor [12]. The antioxidant effect is assessed by monitoring the delay in the kinetic progression of biosensor fluorescence emission [13]. Cells were incubated in fresh serum-free DMEM with a range of sample concentrations for 1 h at 37 °C in a 5% CO_2_ environment. Two independent experiments were performed with the range of sample concentrations, with triplicate wells per concentration (triplicate measurements). Following the 1 h incubation with the samples, the cells were treated with the biosensor for an additional 1 h at 37 °C in 5% CO_2_ using serum-free medium to avoid interactions with serum components. The Relative Fluorescence Unit (RFU) was measured at 535 nm using a Varioskan Flash Spectral Scanning Multimode Reader (Thermo Fisher Scientific, Waltham, MA, USA) following repeated 470 nm LED illuminations applied across the entire 96-well plate. Raw kinetic profiles were recorded during each illumination and fluorescence reading sequence (20 iterations).

The cellular Antioxidant Index (AI) was calculated from normalized kinetic profiles as follows: AI (%) = 100 − 100 (_0_∫^20^ RFU_SC_/_0_∫^20^ RFU_control_), where the control is the cell culture medium only or medium with solvent only. The Antioxidant Index (AI) was plotted against the logarithm of the sample concentration (Log) and fitted to a sigmoid model based on the equation AI = AI_min_ + (AI_max_ − AI_min_)/(1 + 10^(Log(EC50/SC)×HS)^), where SC represents the sample concentration, HS is the Hill slope (the tangent slope at the inflexion point), and EC_50_ is the concentration that achieves 50% of the maximal effect or the half maximal effective concentration. Dose–response curves and the corresponding EC_50_, EC_10_ and EC_90_ were calculated using Prism8 software (GraphPad, San Diego, CA, USA). The best-fit EC_50_ values were determined with 95% confidence intervals using the asymmetrical likelihood method. Coefficients of determination (R^2^) were greater than 0.97 for the calculation of EC values.

### 2.6. Cell Membrane Radical Scavenging Assay with AAPH/DCFH-DA (CAA Assay)

The CAA assay [14] is based on the cell uptake of the DCFH-DA probe (2′,7′-dichlorofluorescein diacetate) through the plasma membrane, being facilitated by its diacetate group (DA). Once inside the cell, the DCFH-DA is hydrolyzed to non-fluorescent DCFH, which becomes fluorescent upon its oxidation to DCF. In our assay, oxidation is initiated by the radical generator AAPH (2,2′-azobis (2-amidinopropane) dihydrochloride), which induces the production of peroxyl radicals at the plasma membrane, converting DCFH to its fluorescent product, DCF. The cells were incubated with concentration ranges of the sample preparations for 1 h at 37 °C in 5% CO_2_ in the presence of DCFH-DA (30 µM). Following incubation, the cells were washed three times, and AAPH was added (600 µM) to initiate radical generation. Two independent experiments were conducted, with each sample concentration tested in triplicate in serum-free culture medium. Fluorescence (RFU) was measured every 5 min in kinetic mode, and the readings continued for the necessary duration. Dose–response curves were calculated using the formula:

CAA Units = 100 − (_0_∫^50^ RFU_sample_/_0_∫^50^ RFU_control_) × 100

### 2.7. ARE–Luciferase Assay

The ability of the samples to activate the Nrf2-regulated ARE pathway was assessed using a luciferase reporter gene assay, as previously described [11]. Stably transfected ARE-luc-HepG2 and ARE-luc-HaCaT cells were incubated for 17 h at 37 °C in 5% CO_2_ with ranges of sample concentrations. Following incubation, the cells were treated with a mixture of cell lysis buffer and luciferin (the substrate of luciferase) (BPS Bioscience, San Diego, CA, USA). Luminescence was measured using a Varioskan Flash Spectral Scanning Multimode Reader (Thermo Fisher Scientific, Waltham, MA, USA) to determine the Relative Luminescence Units (RLUs), reflecting luciferase gene expression via the activation of the ARE sequence. Sulforaphane (SFN) was used as a positive control for the assay. The results were expressed as the fold increase (FI) relative to the negative control at t = 20 min using the formula FI = (RLU_sample_/RLU_control_). FI values were plotted against the logarithm of the sample concentration and fitted to a sigmoid model using the equation FI = FI_min_ + (FI_max_ − FI_min_)/(1 + 10^(Log(EC50-SC)×HS)^), where SC is the sample concentration, HS is the Hill slope, and EC_50_ is the concentration at which 50% of the maximal effect is achieved. Dose–response curves were generated using Prism8 software (GraphPad, San Diego, CA, USA). Two independent experiments were performed, with each sample concentration tested in duplicate. Confidence intervals were calculated using the asymmetrical method, set at 95%. Coefficients of determination (R^2^) were greater than 0.97 for the determination of EC values.

### 2.8. In Vitro Intestinal Absorption Assay

Caco2 cells were cultured at 37 °C in a 5% CO_2_ atmosphere using GlutaMAX-supplemented DMEM complemented with 20% FBS and 1X penicillin–streptomycin. The cells were grown until they reached 70%–80% confluence. For the Transwell experiments, 1.52 × 10^6^ cells/mL were seeded onto 12-well Transwell inserts with polycarbonate membranes (12-well microplate, 12 mm diameter and 3 μm pore size; Corning 3402 (Fisher Scientific SAS, Illkirch, France)). Each well of the Transwell plate had two compartments separated by the membrane: an apical compartment representing the lumen of the small intestine and a basolateral compartment representing the sub-epithelial side. The cells were maintained for 21 days at 37 °C in a 5% CO_2_ incubator, with medium changes in the apical (500 μL) and basolateral (1500 μL) compartments every other day. After 21 days of differentiation, Caco2 monolayers were ready for transepithelial transfer experiments, during which the medium in the apical compartments was replaced with sample preparations. The cells were maintained at 37 °C in a 5% CO_2_ incubator during the transport experiments. Electric resistances (Ω) across the cell monolayers were measured using an ohmmeter (MERS00002 Millipore Voltmeter-Ohmmeter Millicell-ER (Fisher Scientific SAS, Illkirch, France)) before the addition of the sample preparations at time 0. Transepithelial electric resistances (TEERs) were calculated as TEER = [Ω_cell monolayer_ − Ω_filter (cell-free)_] × filter area, where the filter area was 1.131 cm^2^. TEER values above 300 Ω·cm^2^ were considered indicative of Caco2 monolayer integrity. The incubation times for grape pomace extract (at 25 and 6 mg/mL), OenoGrape Advanced Complex (2 mg/mL) and sodium selenite (0.01 and 0.02 mg/mL) were set at 1 h, after which the electrical resistance values were recorded. The change in resistance was expressed as a percentage of the baseline resistance, which was calculated using the following formula: % baseline resistance = ([resistance at exposure time] − [blank insert resistance])/([baseline resistance] − [blank insert resistance]) × 100, where baseline resistance refers to the resistance measured at time 0. Each sample condition was evaluated in duplicate using Transwell inserts. Caco2 cells incubated in a vehicle (either cell culture medium or 4% ethanol) served as the control conditions. Following the transport experiments, the media from basolateral compartments were collected, aliquoted and stored at −20 °C until further analysis in antioxidant bioassays. The recovery of antioxidant activity, or transport efficiency, was calculated using the formula:

Transport % = [estimated concentration in the basolateral fraction]/[concentration in the apical fraction] × 100 × 3.

## 3. Results

To evaluate the intracellular antioxidant efficacy of three selected ingredients—10% lycopene-rich tomato extract, sodium selenite (Na_2_SeO_3_) and grape pomace extract—three distinct cell-based assays were utilized, each targeting different antioxidant mechanisms. These bioassays included the ROS scavenging activity (AOP1 assay), radical scavenging activity at the cell plasma membrane (CAA assay) and activation of the Nrf2-regulated Antioxidant Response Element (ARE) pathway, which was assessed using an ARE-luciferase assay. Efficacy concentrations (EC_50_, EC_10_ and EC_90_) for intracellular antioxidant activities were determined for each assay and ingredient, either individually or in combination as a tryptic mix in the OenoGrape Advanced Complex. Additionally, the study investigated the antioxidant properties of two food supplement formulations, Oenobiol Sun Expert and Solaire Intensif. This analysis was further extended to examine the antioxidant activity of the ingredients and/or their metabolites after their in vitro intestinal absorption using enterocytes, the absorptive cells of the intestinal barrier.

### 3.1. Evaluation of Intracellular ROS Scavenging Activity

#### 3.1.1. Intracellular ROS Scavenging Activity of Ingredients

**Figure 1:**
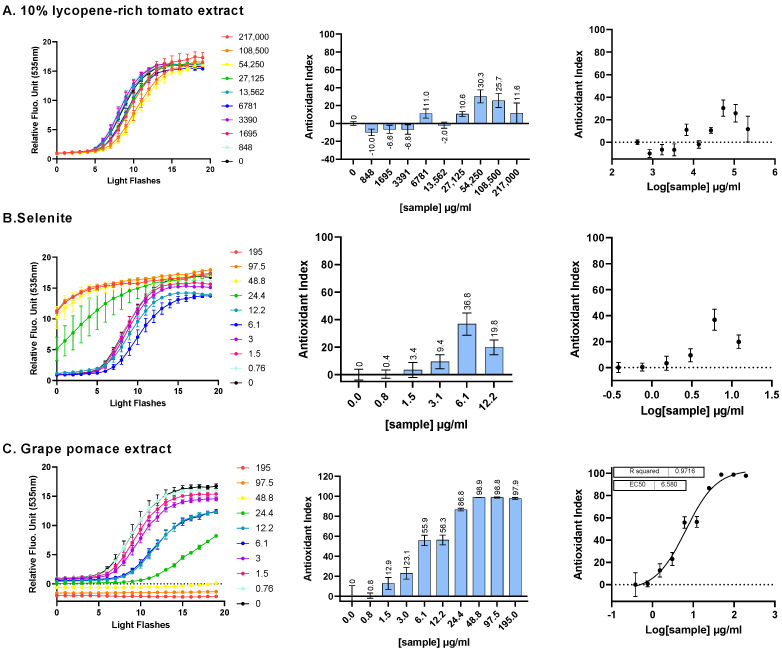
The intracellular ROS scavenging activity of three individual ingredients was assessed on HepG2 cells using the AOP1 bioassay. HepG2 cells were incubated for 1 h with increasing concentrations of a 10% lycopene-rich tomato extract (**A**), sodium selenite (**B**) and grape pomace extract (**C**). Left panel: kinetic fluorescence profiles, where the *x*-axis represents the light flash number, and the *y*-axis displays the Relative Fluorescence Unit (RFU) values for each sample concentration. **Middle panel**: Antioxidant Index (AI) values calculated for each concentration. **Right panel**: dose–response curves with the log-transformed concentration on the *x*-axis and the AI on the *y*-axis. Data points: mean RFU value from triplicate wells; error bars: standard deviation (SD); EC_50_: efficacy concentration required to achieve 50% of the maximum effect; R^2^: coefficient of determination for the dose–response fit.

The intracellular ROS scavenging activity of three ingredients—10% lycopene-rich tomato extract, sodium selenite and grape pomace extract—was assessed individually using the AOP1 bioassay in a dose–response manner on the human hepatocyte HepG2 cell line. The AOP1 assay utilizes recurrent light applications to induce intracellular ROS production via biosensor photoactivation, resulting in an increase in fluorescence. The kinetics of light application and fluorescence measurements were recorded. In the control condition (vehicle, C0) shown in Figure 1, a progressive fluorescence increase was observed over time or with increasing light flashes. Antioxidant activity was indicated by a delay or suppression in the fluorescence increase. The area under the curve (AUC) was used to calculate an Antioxidant Index (AI) for each sample concentration, with AI values ranging from 0 to 100, reflecting the percentage of free radicals neutralized by the sample. For example, in Figure 1C, grape pomace extract at 48.8 µg/mL neutralized 98.9% of the intracellular free radicals. The AI data were fit to a sigmoid model to derive the EC_50_ (efficacy concentration at half-maximum effect), EC_10_ and EC_90_, with the corresponding 95% confidence intervals (CIs) and R^2^ values.

In Figure 1A, increasing concentrations of 10% lycopene-rich tomato extract did not result in a full dose-dependent antioxidant effect, with a maximum AI value of 30.3 observed at 54.25 mg/mL and no EC_50_ value determined (EC_50_ = ND). A similar outcome was noted for sodium selenite (Figure 1B), with a maximum AI of 36.8 at 6.1 µg/mL and no clear dose-dependent profile (EC_50_ = ND), indicating that neither 10% lycopene-rich tomato extract nor selenite exerted a potent antioxidant effect via ROS scavenging. Notably, the AOP1 assay revealed pro-oxidant effects of sodium selenite at concentrations of 24.4 μg/mL (141 μM) or higher, as indicated by fluorescence values exceeding control levels before any light application (Figure 1B, left). In contrast, the grape pomace extract exhibited a full dose-dependent antioxidant response (Figure 1C), with an EC_50_ value of 6.580 µg/mL [95% CI: 4.880–8.649], EC_10_ of 0.9886 µg/mL [0.4081–1.737] and EC_90_ of 43.80 µg/mL [26.32–91.52] (R^2^ = 0.9716), and no evidence of cytotoxicity. Consequently, among the three ingredients tested, the grape pomace extract demonstrated the most potent ROS scavenging activity, exhibiting the highest antioxidant efficacy.

#### 3.1.2. Intracellular ROS Scavenging Activity of OenoGrape Advanced Complex and Oenobiol Formulations

**Figure 2:**
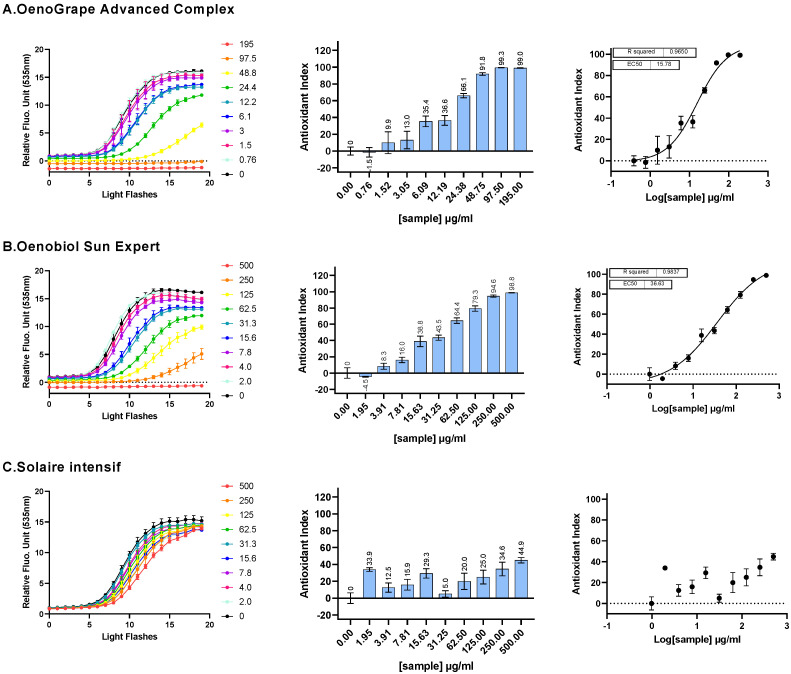
Intracellular ROS scavenging activity on HepG2 cells was assessed for OenoGrape Advanced Complex, Oenobiol Sun Expert and Oenobiol Solaire Intensif formulations. HepG2 cells were incubated for 1 h with increasing concentrations of OenoGrape Advanced Complex (**A**), Oenobiol Sun Expert (**B**) and Oenobiol Solaire Intensif formulations (**C**). **Left panel:** kinetic profile of AOP1 biosensor fluorescence, where the *x*-axis represents light flashes, and the *y*-axis shows relative fluorescence unit (RFU) values recorded for each concentration. **Middle panel:** Antioxidant Index (AI) calculated for each concentration. **Right panel:** dose–response curves, with the *x*-axis representing the log-transformed concentration and the *y*-axis the Antioxidant Index (AI) values. Data points: mean RFU values from triplicate measurements; error bars: SD; EC_50_: efficacy concentration required for 50% efficacity; R^2^: coefficient of determination for the dose–response fit.

The OenoGrape Advanced Complex was made by combining a 10% lycopene-rich tomato extract, sodium selenite and grape pomace extract. The EC_50_ values were determined using the AOP1 bioassay on HepG2 cells. The OenoGrape Advanced Complex demonstrated a dose-dependent antioxidant effect, with an EC_50_ of 15.78 µg/mL [11.57; 22.72] (EC_10_ = 2.303 µg/mL [0.9018; 4.320]; EC_90_ = 108.2 µg/mL [56.52; 364.6]; R^2^ = 0.9650) (Figure 2A). Based on the ratio between the previously measured EC_50_ values of the individual ingredients and the EC_50_ of the OenoGrape Advanced Complex, approximately 41.7% of the antioxidant activity was attributed to the grape pomace extract in the mixture, consistent with the 37.46% (m/m) proportion of grape pomace extract in the complex. This finding indicates the absence of synergistic or antagonistic interactions between the ingredients when combined.

In addition, the ROS scavenging activity of the Oenobiol Sun Expert formulation, measured using the AOP1 assay, also exhibited a dose–response relationship, with an EC_50_ of 36.63 µg/mL [26.68; 58.07] (EC_10_ = 2.250 µg/mL [0.7801; 4.210]; EC_90_ = 596.5 µg/mL [266.3; 2894]; R^2^ = 0.9837) (Figure 2B). Approximately 17.9% of antioxidant activity could be attributed to the grape pomace extract in Oenobiol Sun Expert, which aligns with the 13.3% (m/m) proportion of grape pomace extract in the formulation. In contrast, when Oenobiol Solaire Intensif, which does not contain grape pomace extract, was evaluated using the AOP1 assay, no EC_50_ value could be determined due to the lack of a sigmoid fit (Figure 2C).

In conclusion, the OenoGrape Advanced Complex exhibited potent intracellular ROS scavenging activity, which was also evident in the Oenobiol Sun Expert formulation.

The intracellular ROS scavenging activities measured for the three individual ingredients, the OenoGrape Advanced Complex and the Oenobiol Sun Expert formulation are summarized in Table 1. Notably, both the OenoGrape Advanced Complex and Oenobiol Sun Expert exhibited identical EC_10_ values, which represent the minimum concentrations at which the antioxidant effect is considered significant.

### 3.2. Evaluation of Radical Scavenging Activity at the Cell Membrane

**Figure 3:**
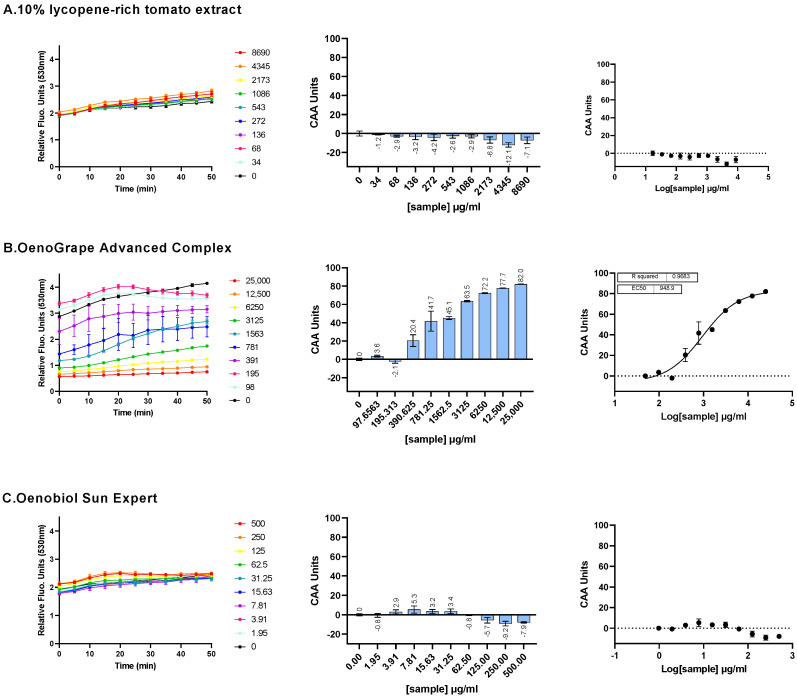
The cell membrane radical scavenging activity of a 10% lycopene-rich extract, the OenoGrape Advanced Complex and Oenobiol Sun Expert formulation was evaluated in HepG2 cells using the CAA or AAPH/DCFH-DA assay. HepG2 cells were incubated for 4 h with varying concentrations of the 10% lycopene-rich tomato extract (**A**), OenoGrape Advanced Complex (**B**) and Oenobiol Sun Expert formulation (**C**). **Left panel:** fluorescence emission kinetics of the DCFH probe. **Middle panel:** Antioxidant Index (AI) calculated for each concentration. **Right panel:** dose–response curves. Data points: mean RFUs of triplicate wells; error bars: SD; EC_50_: efficacy concentrations at which 50% efficacity is observed; R^2^: coefficient of determination for the dose–response fit.

An evaluation of the radical scavenging activity at the cell membrane level was performed using AAPH, a free-radical generating azo compound that acts as an oxidant and initiates lipid peroxidation through the generation of peroxyl and alkoxyl radicals in various membrane systems [15,16]. The ability of a 10% lycopene-rich tomato extract, the OenoGrape Advanced Complex and the Oenobiol Sun Expert formulation to inhibit AAPH-induced ROS generation were assessed in HepG2 cells using the DCFH-DA probe (CAA assay). EC_50_ values could not be determined for the 10% lycopene-rich tomato extract or the Oenobiol Sun Expert formulation, and no antioxidant profiles could be derived (Figure 3A,C). However, for the OenoGrape Advanced Complex, a complete dose–response curve was obtained, with an EC_50_ of 948.9 µg/mL [690.9; 1320] (EC_10_ = 122.6 µg/mL [42.89; 223.8]; EC_90_ = 7343 µg/mL [3825–21,262]) (Figure 3B).

Overall, the EC_50_ value of the OenoGrape Advanced Complex, determined using the CAA assay, was approximately 60 times higher (indicating 60 times lower efficacy) than the value obtained from the AOP1 assay.

### 3.3. Evaluation of the Nrf2-Regulated ARE Induction Activity

#### 3.3.1. ARE Activation by Ingredients

**Figure 4:**
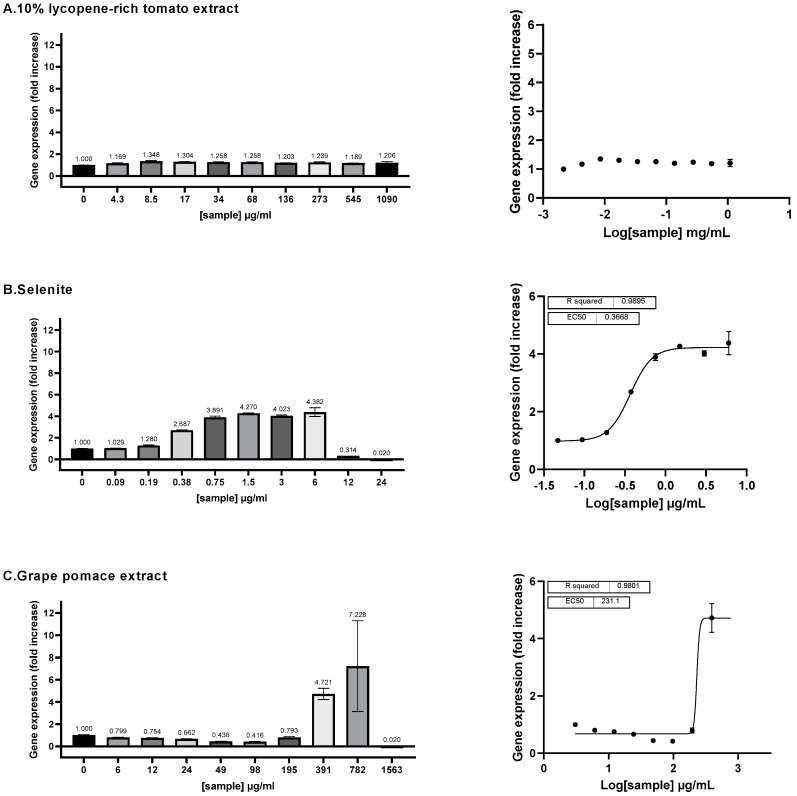
The ARE transcriptional activity of the three individual ingredients was assessed on ARE-luc-HepG2 cells. ARE–luciferase–HepG2 cells were treated for 17 h with a range of concentrations of 10% lycopene-rich tomato extract (**A**), sodium selenite (**B**) and grape pomace extract (**C**), and the luciferase luminescence was measured as relative luminescence units. **Left panel:** the graphs display the luciferase gene expression as the fold increase (FI) relative to the vehicle control. **Right panel**: the dose–response curves are represented, where the log-transformed concentrations are plotted on the *x*-axis against the fold increase in the gene expression (FI). Data points: mean FI of duplicate measurements; error bars: SD; EC_50_: efficacy concentration required for 50% of the maximum effect; R^2^: coefficient of determination for the dose–response curve.

The ability of the three ingredients to activate the Antioxidant Response Element (ARE) pathway was assessed using a HepG2 cell line stably transfected with a luciferase gene under the transcriptional control of the ARE sequence. The grape pomace extract demonstrated a full dose-dependent activation of the ARE pathway, with an EC_50_ of 231 μg/mL (Figure 4C) (EC_10_ = 208.6 μg/mL; EC_90_ = 260.8 μg/mL). The activation effect of grape pomace extract was noticeable, as the luminescence level increased up to 7.328-fold compared to the negative control at 782 μg/mL of grape pomace. At this concentration, the luminescence levels reached 50.3% of the inducing activity observed with 25 μM sulforaphane (SFN), a known potent inducer of the ARE pathway.

Sodium selenite exhibited a strong dose-dependent effect on ARE activation, with an EC_50_ of 0.3668 μg/mL (or 2.15 μM) [0.3281; 0.4094] (EC_10_ = 0.1864 μg/mL [0.1390; 0.2449]; EC_90_ = 0.7219 μg/mL [0.5560; 0.9527]) (Figure 4B). At a concentration of 1.5 μg/mL, sodium selenite induced gene expression up to 4.270 times the basal level. At this concentration, the induction level was equivalent to 30.5% of that achieved by sulforaphane.

In contrast, the 10% lycopene-rich tomato extract did not exhibit any dose–response effect on the activation of the ARE pathway and its EC_50_ could not be determined (Figure 4A).

#### 3.3.2. ARE Activation by OenoGrape Advanced Complex and Oenobiol Formulations

**Figure 5:**
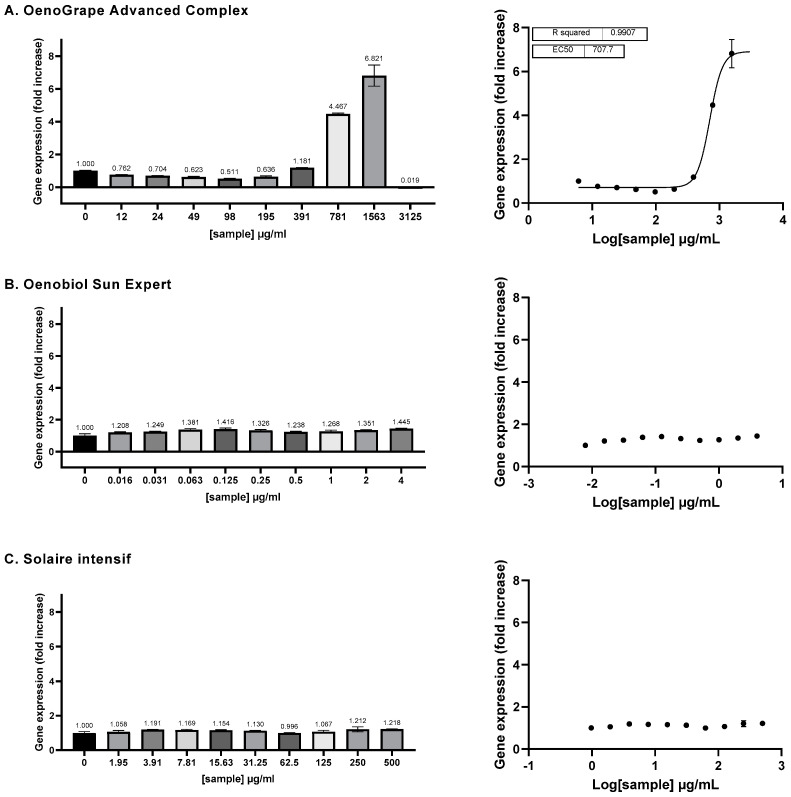
The ARE transcriptional activity of the OenoGrape Advanced Complex, Oenobiol Sun Expert and Solaire Intensif formulations was evaluated on ARE-luc-HepG2 cells. ARE–luciferase–HepG2 cells were treated for 17 h with a range of concentrations of the OenoGrape Advanced Complex (**A**), Oenobiol Sun Expert (**B**) and Solaire Intensif (**C**) formulations, and the luciferase luminescence was measured. The left panel displays the fold increase in gene expression (FI) relative to the vehicle control, while the right panel presents the dose–response curves with the log-transformed concentrations plotted on the *x*-axis and the fold increase in gene expression (FI) on the *y*-axis. Data points: the mean fold increase in gene expression (FI) of duplicate measurements; error bars: SD; EC_50_: efficacy concentration required to achieve 50% of the maximum effect; R^2^: coefficient of determination for the dose–response fit.

The OenoGrape Advanced Complex induced a dose-dependent activation of the ARE pathway, with an EC_50_ of 707.7 μg/mL [655.2; 757.2] (Figure 5A). At a concentration of 1563 µg/mL, the OenoGrape Advanced Complex elicited a maximum fold increase (FI) of 6.8 compared to the vehicle control, corresponding to 47.5% of the activity induced by SFN. However, no dose-dependent activation of the ARE pathway was observed for the two formulations, likely due to the lower concentrations of grape pomace extract and selenite (below their previously determined EC_10_ values) present in these formulations.

### 3.4. Evaluation of Antioxidant Activity Following In Vitro Transepithelial Transport

An evaluation of the bioactivity of samples following in vitro transepithelial transport on enterocytes was conducted to assess whether the intracellular antioxidant activity of the ingredients and the Oenobiol Sun Expert formulation persisted after in vitro intestinal absorption. The compounds were administrated to the apical compartments of differentiated Caco2 cell monolayers grown on Transwell inserts. After 1 h of incubation, the media from the basolateral compartments of the Caco2 cells monolayer were collected and subjected to the AOP1 bioassay using two target cell models: hepatocytes and keratinocytes. Hepatocytes were selected because compounds absorbed via intestinal transport enter the bloodstream and are transported directly to the liver through the portal vein. Keratinocytes were also used as a model, given that the food supplement is intended for skin protection.

#### 3.4.1. Intracellular ROS Scavenging Activity of the Basolateral Fractions from the Caco2 Cells Exposed to Oenobiol Sun Expert and Grape Pomace Extract

Transepithelial electric resistance (TEER) measurements: TEER measurements were conducted to assess the integrity of the Caco2 cell monolayer integrity after exposure to the Oenobiol Sun Expert formulation and grape pomace extract. On day 21, the cells incubated with the cell medium control showed TEER values of 684 Ω·cm^2^ at 0 h and 664 Ω·cm^2^ at 1 h (reflecting a −3% change). For the cells incubated with the solvent control (4% ethanol in cell medium), the TEER values were 614 Ω·cm^2^ at 0 h and 694 Ω·cm^2^ at 1 h (a +13% change), whereas the TEER values were 649 Ω·cm^2^ at 0 h and to 684 Ω·cm^2^ (a +5% change) for the cells exposed to Oenobiol Sun Expert. These TEER values indicated that incubation with 4% ethanol did not damage the cell monolayer integrity within 1 h and that exposure to Oenobiol Sun Expert for 1 h did not affect the TEER values. In contrast, exposure to grape pomace extract at concentrations of 6 and 25 mg/mL resulted in a substantial increase in TEER values, reaching 258% (641.5 to 1659 Ω·cm^2^) and 296% (654 to 1939 Ω·cm^2^), respectively. A dark layer was formed due to grape pomace extract accumulation on the cell monolayer. After washing (on day 24), the TEER was still increased by 144% (774 to 1114 Ω·cm^2^), suggesting that exposure to grape pomace extract may reinforce tight junctions between cells, thereby strengthening the cellular barrier.

**Figure 6:**
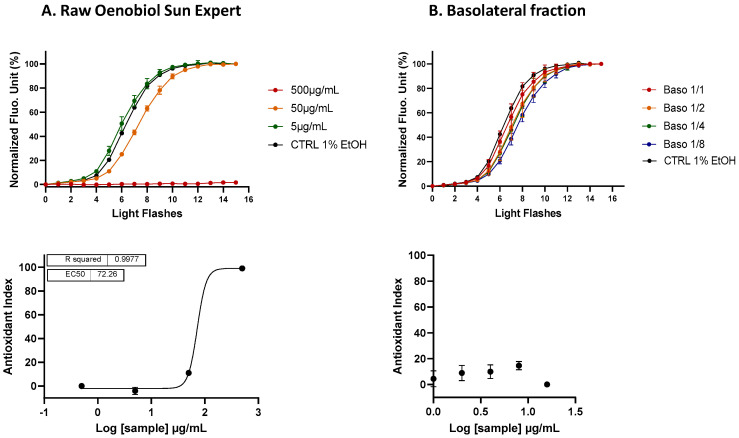
A comparative analysis of the ROS scavenging activity (AOP1 assay) in HepG2 cells was performed for the Oenobiol Sun Expert formulation, before and after intestinal transepithelial transfer. HepG2 cells were treated with increasing concentrations of raw Oenobiol Sun Expert (panel (**A**)) and with serial dilutions of the basolateral fractions obtained from Caco2 cells following a 1 h incubation with Oenobiol Sun Expert (panel (**B**)). Top panel: kinetic fluorescence profiles, with the *x*-axis representing the light flash number and the *y*-axis showing the normalized Relative Fluorescence Unit (RFU) values. Bottom panel: dose–response curves, where log concentrations or log dilutions are plotted on the *x*-axis and the Antioxidant Index on the *y*-axis. Data points: mean RFUs of triplicate measurements; error bars: SD; EC_50_: efficacy concentrations at 50% effect; R^2^: coefficient of determination.

**Intracellular ROS scavenging activity (AOP1 assay) of Caco2 basolateral fractions: study on human liver cells:** Basolateral fractions (1.5 mL) from Caco2 cells exposed to either Oenobiol Sun Expert or grape pomace extract were collected, diluted and, subsequently, subjected to a dose–response analysis using HepG2 cells to perform the antioxidant cell-based assay AOP1. In parallel, raw Oenobiol Sun Expert and grape pomace extract were also tested in the same experiment to estimate the efficiency of antioxidant activity transfer following intestinal absorption. As anticipated, Oenobiol Sun Expert exhibited a full dose–response effect, with an EC_50_ value of 72.43 μg/mL (Figure 6A). However, no measurable antioxidant effect was detected after intestinal passage (Figure 6B). This outcome may be attributed to the limitation imposed by the 4% ethanol content, which restricted the testing of Oenobiol Sun Expert on Caco2 cells at concentrations exceeding 500 µg/mL.

**Figure 7:**
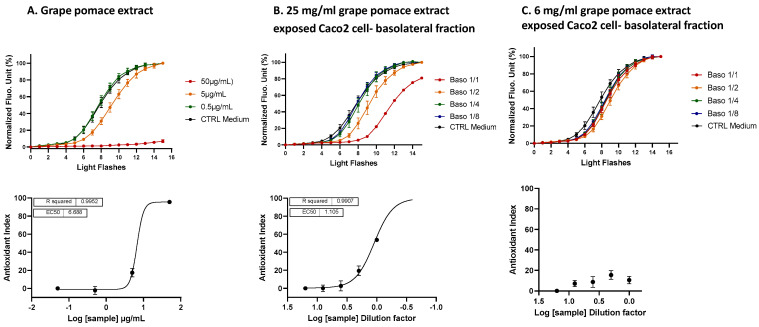
A comparison of the ROS scavenging activity (AOP1 assay) in HepG2 cells was conducted for grape pomace extract before and after intestinal transepithelial transfer. HepG2 cells were treated with increasing concentrations of raw grape pomace extract (panel (**A**)) and increasing dilutions of basolateral fractions collected from Caco2 cells following a 1 h incubation with either 25 mg/mL (panel (**B**)) or 6 mg/mL (panel (**C**)) grape pomace extract. Top panel: kinetic fluorescence profiles, with the light flash number on the *x*-axis and the normalized Relative Fluorescence Unit (RFU) values on the *y*-axis. Bottom panel: dose–response curves, with log concentrations or log dilutions plotted on the *x*-axis and the Antioxidant Index on the *y*-axis. Data points: mean RFUs from triplicate measurements; bars: SD; EC_50_: efficacy concentrations at 50% effect; R^2^: coefficient of determination.

In contrast, grape pomace extract, which was directly solubilized in culture medium, could be administrated to a Transwell Caco2 cell monolayer at higher concentrations (25 mg/mL and 6 mg/mL). Prior to intestinal transfer, the grape pomace extract demonstrated an EC_50_ of 6.711 μg/mL (Figure 7A) and this antioxidant effect was also detectable post-intestinal transport, particularly at the 25 mg/mL concentration, with an EC_50_ established at a dilution factor of 1.105 (Figure 7B). This result indicates the bioavailability of certain compounds and/or metabolites from the grape pomace extract. The bioavailability yield was calculated using the following equation:

Yield % = ([estimated basolateral concentration]_mg/mL_/[apically applied concentration]_mg/mL_) × 3 × 100

where (1) the factor 3 compensates for the volume difference between the apical and basolateral compartments of the Transwell; and (2) the basolateral concentration is estimated based on the Antioxidant Index (AI) value at a 1:1 dilution in the basolateral fraction (Figure 7B), which is then related to the dose–response curve of the grape pomace extract (Figure 7A). From this, the estimated concentration in the basolateral fraction at a 1:1 dilution was 7.1 µg/mL of grape pomace extract (AI = 53.73), resulting in an estimated transfer yield of 0.08%. The basolateral fraction collected after the 6 mg/mL grape pomace treatment showed no antioxidant effect (Figure 7C), which is consistent with the effect in the 1:4 dilution of the basolateral fraction from the 25 mg/mL treatment, as seen in Figure 7B.

**Figure 8:**
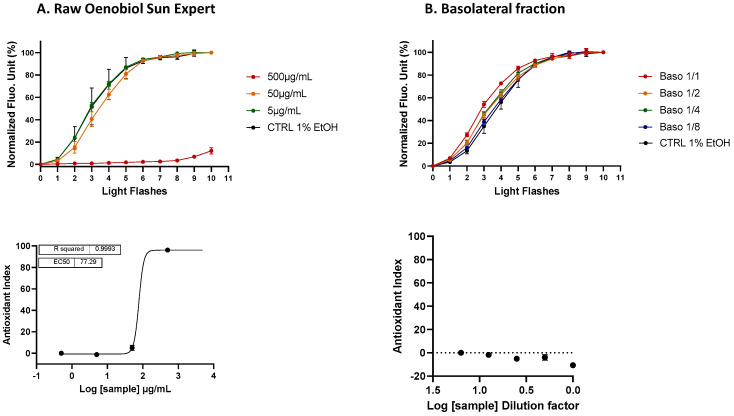
A comparison of the ROS scavenging activity (AOP1 assay) in HaCaT cells was performed for the Oenobiol Sun Expert formulation before and after intestinal transepithelial transfer. HaCaT cells were treated with increasing concentrations of raw Oenobiol Sun Expert (panel (**A**)) and increasing dilutions of basolateral fractions obtained from Caco2 cells after a 1 h incubation with Oenobiol Sun Expert (panel (**B**)). Top panel: kinetic fluorescence profiles, with the light flash number on the *x*-axis and the normalized Relative Fluorescence Unit (RFU) values on the *y*-axis. Bottom panel: dose–response curves, with log concentrations or log dilutions plotted on the *x*-axis and the Antioxidant Index on the *y*-axis. Data points: mean RFUs of triplicate measurements; bars: SD; EC_50_: efficacy concentrations at 50% effect; R^2^: coefficient of determination.

**Intracellular ROS scavenging activity (AOP1 assay) of basolateral fractions: study on human keratinocytes:** The antioxidant activities of Oenobiol Sun Expert and grape pomace extract were evaluated using the AOP1 assay on HaCaT cells. Raw Oenobiol Sun Expert demonstrated potent antioxidant activity, with an EC_50_ of 77.15 μg/mL (Figure 8A), a value closely aligned with the EC_50_ previously measured on hepatocytes. However, this antioxidant activity was not observed in the basolateral fractions of Caco2 cells exposed to 2 mg/mL of the Oenobiol Sun Expert formulation (Figure 8B). This lack of detection may again be attributed to the concentration range limitation imposed by the presence of the solvent (ethanol), which restricted the maximum testable concentrations on Caco2 cells.

**Figure 9:**
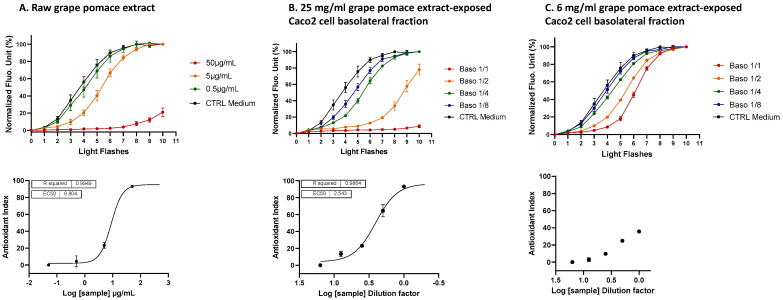
A comparison of the ROS scavenging activity (AOP1 test) was conducted on HacaT cells for grape pomace extract before and after intestinal transepithelial transfer. HaCaT cells were treated with increasing concentrations of raw grape pomace extract (panel (**A**)) and increasing dilutions of basolateral fractions from Caco2 cells after a 1 h incubation with 25 mg/mL (panel (**B**)) or 6 mg/mL (panel (**C**)) of grape pomace extract. Top panel: kinetic fluorescence profiles, with the light flash number on the *x*-axis and the normalized Relative Fluorescence Unit (RFU) values on the *y*-axis. Bottom panel: dose–response curves with log concentrations or log dilutions plotted on the *x*-axis and the Antioxidant Index on the *y*-axis. Data points: mean RFUs of triplicate measurements; bars: SD; EC_50_: efficacy concentrations at 50% effect; R^2^: coefficient of determination.

In contrast, grape pomace demonstrated efficient antioxidant activity in HaCaT cells, with an EC_50_ of 8.803 μg/mL (Figure 9A). The basolateral fraction collected from the 25 mg/mL grape pomace extract treatment displayed a full dose response, with an EC_50_ established at a dilution factor of 2.54 (Figure 9B). Using the previously mentioned calculation method, the transfer yield was estimated at 0.63%, which is eight times higher than the yield calculated from hepatocytes. Notably, the basolateral fraction from the 6 mg/mL treatment condition, a concentration similar to that of grape pomace extract in the Oenobiol Sun Expert formulation, also exhibited a significant antioxidant effect (Figure 9C). This result is consistent with the antioxidant effect observed with the 1:4 dilution of the basolateral fraction from the 25 mg/mL treatment condition (Figure 9B).

#### 3.4.2. ARE Induction Activity by the Basolateral Fractions from Caco2 Cells Exposed to Sodium Selenite

Two concentrations of sodium selenite, 20 μg/mL and 10 μg/mL, which are close to the 16.6 μg/mL present in Oenobiol Sun Expert, were administrated to Caco2 cells for 1 h. Following incubation, the basolateral fractions were collected to assess their ability to induce ARE transcription. This evaluation was conducted using the ARE–luciferase test performed on stably transfected hepatocytes and keratinocytes.

**Transepithelial electric resistance (TEER) measurements:** On day 21, the TEER values of Caco2 cells were measured before and after a 1 h exposure to sodium selenite. A 43.47% decrease in TEER was observed in the 20 μg/mL selenite condition, with values dropping from 644 to 364 Ω·cm^2^. Similarly, a 33.33% reduction was recorded for the 10 μg/mL selenite condition, where the TEER values decreased from 711 to 474 Ω·cm^2^. A comparable decrease of 34.98% was observed in the sulforaphane (4 µg/mL SFN) treatment condition, with TEER values declining from 729 to 474 Ω·cm^2^. Despite the reductions, these TEER values remain indicative of a functional intestinal epithelium.

**Figure 10:**
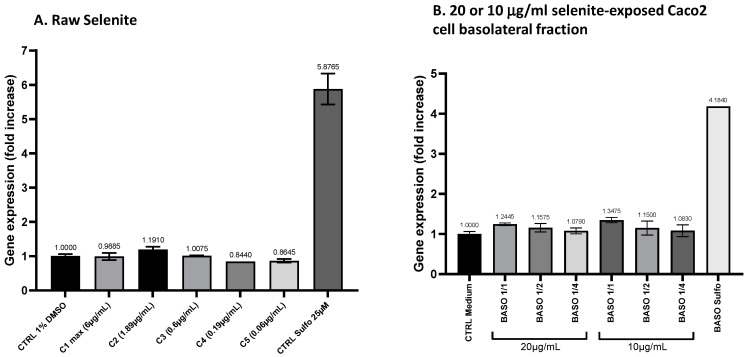
A comparison of ARE transcriptional activity comparison in ARE–luciferase–HepG2 cells was conducted for sodium selenite before and after intestinal transfer. ARE–luciferase–HepG2 cells were treated for 17 h with a range of sodium selenite concentrations (panel (**A**)) or dilutions of basolateral compartments collected from Caco2 cells after a 1 h incubation with 10 or 20 μg/mL sodium selenite (panel (**B**)). Luciferase luminescence was measured as an indicator of ARE pathway activation. The graphs depict the gene expression fold increase (FI) relative to the vehicle control for either decreasing concentrations of sodium selenite or varying dilutions of the basolateral fractions. Data points: mean fold increase (FI) of duplicate measurements; bars: SD.

**ARE induction activity by Caco2 basolateral fractions: study on hepatocytes:** Basolateral fractions from Caco2 cells exposed to 10 or 20 μg/mL sodium selenite for 1 h were collected and diluted (1:1; 1:2; 1:4) for the HepG2–ARE–luciferase assay. Similarly, basolateral fractions from Caco2 cells treated with sulforaphane were collected and assessed. After intestinal transport, sodium selenite was able to activate the ARE pathway on HepG2 cells. Although the EC_50_ could not be determined, the undiluted (1:1) basolateral fractions from Caco2 cells treated with 0.01 or 0.02 mg/mL sodium selenite showed increases in transcriptional activity compared to the vehicle control. This increase corresponded to a 1.35-fold increase (FI) in gene expression for the 0.01 mg/mL treatment and a 1.24-fold increase for the 0.02 mg/mL treatment. For comparison, sulforaphane (SFN) exposure under the same conditions resulted in a 4.18-fold increase in transcriptional activity (Figure 10).

**Figure 11:**
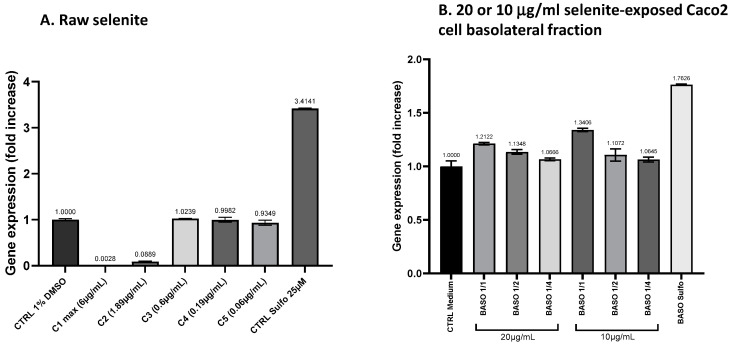
A comparison of ARE transcriptional activity in ARE–luciferase–HacaT cells was conducted for sodium selenite before and after intestinal transfer. ARE–luciferase–HacaT cells were treated for 17 h with a range of concentrations of sodium selenite (panel (**A**)) or dilutions of basolateral fractions collected from Caco2 cells following a 1 h incubation with 10 or 20 μg/mL sodium selenite (panel (**B**)). Luciferase luminescence was measured to assess ARE pathway activation. The graphs display the fold increase (FI) in gene expression compared to the vehicle control for either decreasing concentrations of sodium selenite or different dilutions of the basolateral fractions. Data points: mean fold increase (FI) of duplicate measurements; bars: SD.

**ARE induction activity by Caco2 basolateral fractions: study on keratinocytes:** In the keratinocyte model, the undiluted (1:1) basolateral fractions from Caco2 cells exposed to 0.01 and 0.02 mg/mL sodium selenite resulted in ARE transcriptional activity with fold increases (FIs) of 1.34 and 1.21, respectively (Figure 11B). This indicates that sodium selenite, following intestinal transfer, retains its ability to moderately activate the ARE pathway in keratinocytes.

These results demonstrate that sodium selenite, or its metabolites, retained the ability to act as Nrf2-regulated ARE inducers in both hepatocytes and keratinocytes after intestinal transfer. Notably, this induction appeared to be enhanced following intestinal absorption, especially in keratinocytes. Prior to absorption, the effect of selenite was approximately 20% of the sulforaphane (SFN)-induced activity in HepG2 cells and 30% in HaCaT cells. Following intestinal absorption, selenite’s effect (and/or of its metabolites) increased to around 30% of SFN activity in HepG2 cells and approximately 70% in HaCaT cells.

## 4. Discussion

In this study, we demonstrated that two components of the OenoGrape Advanced Complex, grape pomace extract and sodium selenite, exhibited effective and complementary intracellular antioxidant activities, while the 10% lycopene-rich tomato extract did not contribute to these antioxidant effects. Grape pomace extract showed high ROS scavenging activity, while sodium selenite functioned as a strong ARE pathway inducer, both contributing to enhanced cellular protection. The high ROS scavenging activity persisted in the final formulation of the food supplement. Furthermore, by assessing the antioxidant activity following in vitro intestinal barrier transport of the ingredients, grape pomace extract and selenite were shown to be bioavailable and capable of executing biological functions on two target cell models.

**Lycopene-rich tomato extract:** In our study, no dose-dependent antioxidant activity was observed for the 10% lycopene-rich tomato extract in any of the three cell-based antioxidant mechanisms examined (AOP1, CAA and ARE–luciferase assays). In the skin, carotenoids, including lycopene, accumulate primarily in the epidermis, where they serve as a protective barrier against environmental factors such as free radicals and UV radiation [17,18]. Carotenoids are known for their photoprotective effects through direct light absorption and antioxidant properties [19]. Lycopene was shown to act via the neutralization of radicals or singlet oxygen (1O2) in organic solvents and cell-free systems [20,21]. However, in complex cell systems, carotenoids appear to act differently. Using cell lines, Bosio et al. [22] demonstrated that intracellular β-carotene did not scavenge singlet oxygen (1O2). Other authors suggested that carotenoids might even act as pro-oxidants [23,24]. In our study, no pro-oxidative nor scavenging antioxidative effects were observed for the 10% lycopene-rich tomato extract assessed in a dose-dependent manner. Additionally, the 10% lycopene-rich tomato extract did not activate the Nrf2-regulated ARE pathway. Carotenoids can act indirectly via the modulation of stress-dependent signaling pathways [25]. Ben-Dor et al. [26] reported that a lycopene ethanolic extract was able to induce an ARE transcription system in transiently transfected mammary cells. However, this extract contained hydrophilic derivatives, and the authors concluded that some lycopene oxidation products were the active mediators in this activation [27]. Indeed, Lian & Wang demonstrated that enzymatic metabolites of lycopene in mammalian tissues, called apo-10′-lycopenoids (lycopenals, lycopenols and lycopenoic acids), were responsible for the Nrf2 nuclear accumulation and antioxidant protein expression in human bronchial epithelial cells [28].

**Sodium selenite:** Our results indicated that sodium selenite does not exert direct ROS scavenging activity. Selenium, which is ingested through the human diet in a few chemical forms (inorganic forms as selenate and selenite; organic forms as mainly selenomethionine), plays a biological role as a component of selenoproteins (with selenocysteine being referred as to the “21st” amino acid). In humans, 25 genes coding for selenoproteins have been identified [29]. Many of these selenoproteins regulate oxidative stress and antioxidant defense (e.g., six glutathione peroxidases (GPxs) and three thioredoxin reductases (TrxR)) and other biological functions, such as thyroid hormone metabolism and immune and inflammatory responses [30]. While selenium contributes to cellular antioxidant mechanisms through selenoproteins, it does not act as a direct ROS/RNS scavenger. In our AOP1 study, we clearly demonstrated that sodium selenite (1) had no direct ROS scavenging action and (2) that sodium selenite exhibited a pro-oxidative effect at high concentrations (24.4 μg/mL or higher selenite concentrations) (141 μM) after only 1 h of exposition, whereas the lower concentrations (0.8 μg/mL–12.2 μg/mL) remained without noticeable effects. Excessive selenium intake can result in toxicity, potentially generating oxidative stress, while selenium deficiencies from insufficiently low daily intake or intestinal absorption defects have also been described [31,32]. The administration of supra-physiological concentrations of sodium selenite for 24 h to endothelial cells induced endoplasmic reticulum stress and increased ROS production, leading to endothelial dysfunction [33]. Sodium selenite is reduced to selenide via glutathione-dependent reactions [34], and subsequently, selenite may exert its toxicity through the consumption of intracellular glutathione, resulting in severe oxidative stress. In our study, we also demonstrated that sodium selenite was a strong ARE pathway inducer at sub-microgram/mL levels. The efficacy concentrations of an ARE-inducing effect of selenite were determined (ARE EC_10_ = 0.186 mg/mL, ARE EC_50_ = 0.367 mg/mL, and ARE EC_90_ = 0.722 mg/mL) and these values were compared to the optimal plasma concentration range for selenium, reported as 0.086 μg/mL to 0.258 μg/mL [35]. Notably, the EC10 value of sodium selenite, representing the minimal selenium concentration to act as an ARE inducer, was within the physiological range of selenium plasma concentrations. Recently, the addition of a selenium-based supplement to the culture medium of rat mesenchymal stem cells from bone marrow was shown to improve both the cytoprotection and stemness capacity (e.g., viability and differentiation potential) of these cells [36]. Also, Ma et al. [37] demonstrated, in vivo and in vitro, that selenium reduced oxidative stress and the release of pro-inflammatory cytokines in rats and in vascular endothelial cells (HUVEC cells) exposed to toxic silver nanoparticles.

Furthermore, in our study, the ARE-inducing activity of selenite (and/or its metabolites) was detected both directly and after intestinal transepithelial transfer experiments. Intestinal absorption mechanisms differ depending on the chemical form of selenium. Selenite absorption was reported to occur by passive diffusion or utilizing amino acid transport systems (through formation of selenocysteine (SeCys) and selenoglutathione (SeGSH), transported across enterocytes by amino acid or peptide transporters). Some experiments of selenium uptake, performed on isolated brush-border membrane vesicles (BBMVs) from rats fed with diets comprising different selenium forms, indicated a very high accumulation of selenite in the rat BBMVs, even higher than the accumulations measured for SeMet and selenate [38,39]. For intestinal transport, Lu Wang and Fu [28] evaluated the transport efficiency of sodium selenite in the Caco2 cell monolayer to be about 2%, which was lower than the transport efficiency of SeMet (5%). Also, the selenium bioavailability from selenite-enriched lettuce was assessed in a vegetable biofortification study, in which in vitro simulated gastrointestinal digestion was followed by Caco2 cell experiments, and selenite transport was estimated to be about 18% [40]. In summary, sodium selenite appeared to be absorbed efficiently, and our study suggests that selenite and/or its metabolites exert ARE-inducing activity.

**Grape pomace extract:** Grape pomace is a by-product of the wine industry and consists of skins, seeds and stems. It is particularly rich in antioxidant polyphenols, which includes flavonoids—such as anthocyanins, flavonols and flavanols—and non-flavonoids, like stilbenes and phenolic acids [41]. The anthocyanins present in grape pomace are glycosylated derivatives of cyanidin, malvidin, delphinidin and peonidin, while the flavonols include forms of quercetin, myricetin, rutin and kaempferol. The flavanols are represented by (+)- catechin and (-)-epicatechin. The stilbenes, well known as being associated with grapes, are *trans*-resveratrol and viniferins. Phenolic acids, including hydroxybenzoic acids (gallic acid) and hydroxycinnamic acids (ferulic, p-coumaric and caffeic acids) also contribute to the potent antioxidant properties of grape pomace. In our study, grape pomace confirmed important intracellular antioxidant properties, with this extract showing highly potent intracellular ROS scavenging activity (AOP1 EC_50_ = 6.80 μg/mL), likely due to the synergistic action of its bioactive compounds. For a comparison with the results obtained for pure polyphenolic compounds assessed using the AOP1 bioassay, Gironde et al. [13] reported AOP1 EC_50_ values of 7.15 μg/mL for quercetin, 14.75 μg/mL for resveratrol, 161.3 μg/mL for catechin and 181.3 μg/mL for epicatechin in HepG2 hepatocytes. Similarly, Furger et al. [11] evaluated six grape extracts (seed, fruit pomace and leaf) in intestinal cells using the AOP1 assay, finding EC_50_ values between 11.62 to 162.2 μg/mL, with the highest activities observed in seed extracts, followed by fruit pomace and leaf extracts. The OenoGrape Advanced Complex, composed of the grape pomace (skin, seed and stem) exhibited superior intracellular ROS scavenging efficacy compared to other grape extracts tested. Resveratrol, one of the key polyphenols in grapes, has been shown since the 1990s to function as an antioxidant, inhibiting free radical formation in a dose-dependent manner in TPA-treated HL-60 cells [42]. Additionally, resveratrol activated the Nrf2-mediated ARE pathway by inducing phase II detoxification enzymes [43]. In our study, the grape pomace extract activated the ARE pathway with an EC_50_ value of 231 μg/mL. Furger et al. [11] observed no Nrf2-regulated ARE activity in two grape seed extracts that displayed the highest AOP1 EC_50_ values, whereas the grape leaf extract showed an ARE EC_50_ of 661 μg/mL. Our previous studies in hepatocytes [44] found that quercetin (EC_50_ = 5.26 μg/mL) and caffeic acid (EC_50_ = 46 μg/mL) were effective inducers of the Nrf2/ARE pathway, as was resveratrol (with EC_50_ = 33.55 μg/mL, unpublished data). Moreover, Soeur et al. [45] demonstrated that resveratrol enhanced Nrf2 nuclear accumulation and induced the expression of ARE-regulated genes and proteins in primary human keratinocytes (NHKs). They further showed that resveratrol, at concentrations between 20 and 100 μM, could induce Nrf2-ARE genes in a full-thickness reconstructed human skin model and increased cellular glutathione (GSH) levels. Kim et al. [46] reported that the pretreatment of HaCaT cells with 625 μg/mL of grape peel extract provided protection against UV-induced cell damage by increasing cytoplasmic heme oxygenase-1 (HO-1) and nuclear Nrf2 protein levels. Additionally, oral supplementation with grape peel extract (1 or 2 g/kg body weight) or resveratrol (2, 10 or 50 mg/kg body weight) for six weeks before UVB exposure reduced wrinkle formation in murine skin. However, the mechanisms by which resveratrol accumulates in the dermis and its effective concentration for exerting antioxidant effects in the skin remain to be elucidated.

The bioavailability of polyphenols varies widely between classes, but in general, dietary polyphenols are poorly absorbed and extensively metabolized in enterocytes and then in the liver through phase I and II reactions. Furthermore, the microbial metabolites generated in the colon significantly contribute to the biological effects of these compounds [47,48]. In our study, polyphenol metabolites from pomace grape extract, produced during in vitro Caco2 cell transport, still exhibited direct ROS scavenging activity, adding complexity to the paradox of “low bioavailability, high bioactivity”.

## 5. Conclusions

Grape pomace extract has demonstrated both direct ROS scavenging activity and indirect antioxidant activity via the activation of the ARE pathway. The ROS scavenging activity of grape pomace, as measured using the AOP1 assay, yielded an EC_50_ of 6.80 μg/mL, while its ARE-inducing capacity showed an EC_50_ of 231 μg/mL. Sodium selenite exhibited a notably strong induction of the ARE pathway, with an EC_50_ of 0.3668 μg/mL. The 10% lycopene-rich tomato extract showed no antioxidant activity in any of the three cell-based assays used in this study. Importantly, when combined in the OenoGrape Advanced Complex, the mixture of grape pomace extract, sodium selenite and tomato extract maintained both direct and indirect antioxidant activities. The AOP1 EC_50_ for the OenoGrape Advanced Complex was 15.78 μg/mL, while the ARE EC_50_ was 707.7 μg/mL. Additionally, the Oenobiol Sun Expert formulation containing the complex also retained potent direct ROS scavenging activity (AOP1 EC_50_ = 36.63 μg/mL). Moreover, the compounds and metabolites present in the basolateral compartment following the in vitro intestinal transfer of grape pomace extract and sodium selenite displayed direct and indirect antioxidant activities on both hepatocytes and keratinocytes. The antioxidant efficacy, as reflected by the ROS scavenging activity, was calculated as 0.08% for HepG2 cells and 0.63% for HaCaT cells, providing insights into the relative bioactivities of these compounds post-intestinal absorption. This study highlights the ability of grape pomace extract and sodium selenite to maintain both direct and indirect antioxidant activities through different mechanisms and underscores their utility in food supplement formulations.

## Figures and Tables

**Table 1:** Efficacy concentrations for intracellular ROS scavenging activity of the three ingredients, OenoGrape Advanced Complex and Oenobiol Sun Expert were determined using the AOP1 assay in HepG2 cells. EC_10_, EC_50_ and EC_90_: concentrations required to achieve 10%, 50% and 90% of the maximal antioxidant activity; 95% CI: 95% confidence interval; R^2^: coefficient of determination for the dose–response fits; ND: values not determined.

Tested Compounds	EC_10_ (μg/mL) [95% CI]	EC_50_ (μg/mL) [95% CI]	EC_90_ (μg/mL) [95% CI]	R^2^
10% lycopene-rich tomato extract	ND	ND	ND	ND
Sodium selenite	ND	ND	ND	ND
Grape pomace extract	0.9886 [0.4081; 1.737]	6.580 [4.880; 8.649]	43.80 [26.32; 91.52]	0.9916
OenoGrape Advanced Complex	2.303 [0.9018; 4.320]	15.78 [11.57; 22.72]	108.2 [56.52; 364.6]	0.9650
Oenobiol Sun Expert	2.250 [0.7801; 4.210]	36.63 [26.68; 58.07]	596.5 [266.3; 2894]	0.9837

## Data Availability

The data presented in this study are available on request from the corresponding authors.
